# Enhanced oil recovery by using modified ZnO nanocomposites in sandstone oil reservoirs

**DOI:** 10.1038/s41598-024-53138-5

**Published:** 2024-02-02

**Authors:** Yaser Ahmadi, Mohsen Mansouri, Peyman Pourafshary

**Affiliations:** 1 Chemical and Petroleum Engineering Department, Ilam University, P.O. Box 69315/516, Ilam, Iran; 2 School of Mining and Geosciences, Nazarbayev University, Astana, Kazakhstan

**Keywords:** Chemistry, Engineering

## Abstract

Recently, nanocomposites were employed to improve the extraction of oil in different reservoirs. Due to the unique characteristics of nanoparticles such as small size, efficient altering main mechanisms such as IFT, CA, and viscosity reduction, have received wide attention among researchers. This study investigated the application of a newly designed ZnO-cerium N-composite for EOR at reservoir conditions, and the performance was compared to the standalone ZnO nanoparticles. After performing the morphology of the N-composite, the effect of the N-composites on the wettability alteration, interfacial tension, viscosity, Zeta potential, pH, and density was studied at different N-composites concentrations at reservoir conditions. Based on the results of rock/fluid interactions at the static phase, an optimum concentration was chosen for performing dynamic core flooding experiments. At 100 ppm, the highest stability and the highest reduction in capillary force were observed. The presence of Ce in the structure of the N-composite changes the pore volume of ZnO-Ce compared to ZnO nanoparticles, which affects the surface charge. IFT (mN/m), CA (°), and zeta potential (mV) were (22.51, 40.83, and − 44.36), and (30.50, 50.21, and − 31.05) for ZnO-Ce and ZnO, respectively at 100 ppm. By application of the optimized nanofluid in an oil displacement study, RF in the presence of ZnO-Ce, and ZnO were 37.11% and 71.40%, respectively.

## Introduction

Oil production from existing oilfields is dropping due to reservoir depletion. Different secondary and tertiary oil recovery methods are used to improve oil production. As an effective technique, chemical enhanced oil recovery (CEOR) methods are used indifferent projects worldwide. CEOR includes injecting various chemicals such as polymer, surfactant, and alkaline^1–6^. These chemicals are used for increasing the oil recovery by changing the effective parameters of capillary forces such as interfacial tension and wettability. Two main challenges in the CEOR approach are the cost of chemicals and high chemical loss in the porous media due to adsorption and trapping^7,8^. So, novel, efficient, and cheaper CEOR methods are being developed through the application of nanotechnology. Because of their small size and high surface-to-volume ratio, nanofluids are being researched as novel CEOR agents^9–11^. Kumar et al.^11^ used SiO_2_-HPAM for IFT reduction, and wettability alteration. CaCl_2_ (1000 mg/L), and NaCl (30,000 mg/L) at 80 °C and 25 °C were used, and it was found that reduction of interfacial tension, and contact angle were higher in the presence of SiO_2_-HPAM in compared to SiO_2_, and presented material was suggested for CEOR^10^. Lashari et al.^12^ used HPAM/GO-SiO_2_ for wettability alteration. At 0.05 wt. % of nanocomposites, the percentage of incremental oil recovery was raised to 30.82%^11^.

The injection of nanofluids alters the sweep efficiency in reservoirs by mechanisms such as wettability alteration and interfacial tension reduction^13^. The high surface energy of the nano-structures provides exceptional electrical, rheological, thermal, and interfacial performance^14,15^. ZnO/SiO_2_/Xanthan was employed by Ahmadi et al.^15^ to reduce wettability and interfacial at varying salinities. The findings showed that at 2 SW (sea water) and 10 SW, the contact angle fell from (79°, 75°) to (78°, 74°) and (70°, 62°), respectively. Therefore, due to their high surface area and small size, nanoparticles can easily pass through porous media and pore throats^16^. Different types of nanoparticles have been used to improve oil recovery, such as silica^17^, ZnO^18^, silica/graphene^19^, carbon^20,21^, kaolinite^22^, and metal oxide^23^ through different mechanisms such as viscosity, IFT, and contact angle reduction. Taborda et al.^17^ used Silica nanoparticles, and it was found that viscosity reduced from 12 to 45%. ZnO with two distinct diameters, 55.7 and 117.1 nm, was employed by Adil et al.^18^ for EOR on sand packs ranging in size from 265 to 300 mD at 95 °C and 0.1 weight percent concentration. In the presence of ZnO, a tertiary recovery of 9–10.4% was noted. Additionally, silica/grapheme was utilized by Afzalitabar et al.^19^ for IFT and contact angle reduction. Their findings showed that the IFT dropped from 53.90 to 29.82 mN/m and the contact angle decreased from 143.47° to 58.50°.

Multiple mechanisms are at play during the flooding of nanoparticles to enhance oil detachment. For instance, the presence of nanoparticles causes a layer to form between the oil and water during flooding via a porous media, which lowers the interfacial tension^9,12^. In certain circumstances, variations in viscosity, wettability, and disjoining pressure are also believed to be helpful mechanisms.

Small ZnO nanoparticles, ranging in size from 10 to 100 nm, have been utilized in CEOR and documented in multiple publications. ZnO/SiO_2_ nanocomposites were employed for EOR in the Kurdistan area of Iraq by Ali et al. in 2019. IFT dropped from 31.8 to 2.016 mN/m, and RF increased from 46.96 to 66.24% at 2000 ppm of nanocomposites. Additionally, the presence of nanocomposites caused the relative permeability intersection to move to the right and the rock's wettability to transition from oil to water^24^. The industry finds zinc oxide nanoparticles appealing due to their low toxicity and biocompatibility^25,26^. ZnO nanoparticles have impact on wettability and IFT in the interactions between oil, brine, and rock. In the presence of ZnO, various trends in IFT change have been noted. Soleimani et al.^27^ found that ZnO nanofluid flooding increased IFT, but Zaid et al.^28^ revealed that IFT decreased when in contact with ZnO nanoparticles. Rezk et al.^29^ also confirmed that the primary causes of an observed 8% incremental oil recovery by ZnO nanofluid flooding are IFT reduction and wettability alteration. ZnO nanoparticles at the oil/brine interface have been shown by Ogolo et al.^30^ to boost oil recovery to 50%. When Adil et al.^31^ looked at how the ZnO concentration affected the recovery factor, they found that the best conditions were at 0.1% and 55.7 nm nanoparticle size, which led to a 10.2% increase in recovery. High ZnO adsorption on calcite sheets was reported by Soleimani et al.^32^, leading to changes in wettability and an 11.8% increase in oil recovery with flooding of 0.3 weight percent ZnO nanofluid.

Researchers have recently devised a novel method termed "nanocomposites-assisted CEOR applications" to boost the effectiveness of nanoparticles like ZnO. The concept underlying nanocomposites involved combining various solid materials to enhance their functionality at the nanoscale over stand-alone applications. Using nanocomposites resulted in efficient mechanisms such as wettability modification, capillary pressure decrease, and the creation of more stable nanofluids. Recently, our team synthesized different nanoparticles for CEOR applications at reservoir conditions. It was found that nanoparticles that contain ZnO in combination with other nanoparticles such as SiO_2_, zirconia, nickel, and alumina are effective in reducing the capillary pressure in porous media and controlling the asphaltene adsorption on rock surfaces^33,34^. Jafarbeigi et al.^33^ showed that a combination of ZnO-alumina nanoparticles has an effective influence on increased oil recovery, wettability modification, and IFT reduction. At 300 ppm, IFT and CA decreased from (27.24 mN/m, 161°) to (11.00 mN/m, 31°). Ahmadi et al.^34^ used alumina-ZnO-urea nanocomposites for improving SWAG tests as a new EOR method at reservoir conditions. It was observed that, at the SWAG ratio of 1:1, and 40 °C, oil recovery was increased from 55.9 to 83.1% through the injection of 80 ppm nanofluid. Ahmadi et al.^35^ also showed that cerium nanoparticles (cerium-zeolite-zirconia) increased the recovery factor from 42.5 to 72% during nanocomposites-assisted WAG tests due to their high total surface area (331.67 m^2^/g). Recently, Mehrooz et al.^36^ used different concentrations of cerium oxide nanoparticles and observed that with 0.01, 0.1, 0.25 and 0.5 wt.% nanofluid, the oil recovery increased to 25%, 38%, 43% and 45%, respectively.

In this study, ZnO nanoparticles were modified with cerium to improve the performance of the nanofluid in changing the interface properties. Different characterization tests such as XRD, BET, EDX, SEM, TEM, DLS, and FT-IR were used to optimize the nanocomposites concentration (OPC) by using Zeta potential and CA testing in reservoir settings which were not considered yet based on our knowledge. After selecting an OPC, the oil displacements were investigated between ZnO-Ce and ZnO. All experiments were obtained at reservoir conditions.

## Materials and methodology

### Material

**Figure 1:**
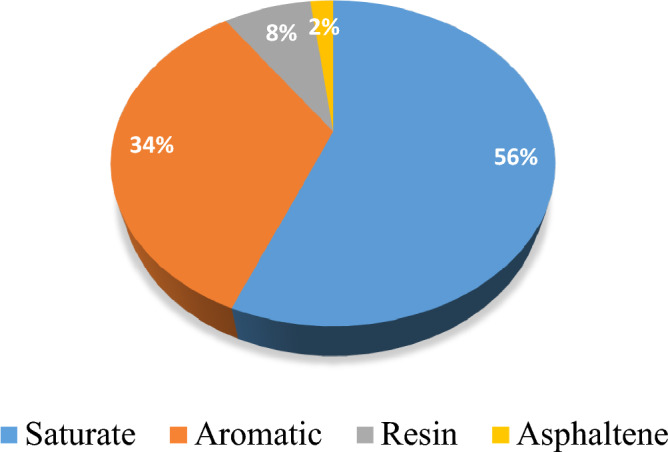
Compositional specifications of the oil.

A sandstone reservoir in Iran provided the crude oil and core. The density, API, and crude oil viscosity at a reservoir pressure of 1800 Psi and temperature of 60 °C are 0.846 cm/g, 28, and 9.9 cP, respectively. Core porosity, core permeability, and GOR were found to be 22.5%, 27.45 mD, 240 scf/STB, and 232, in that order. Figure 1 displays the SARA (saturate-aromatic-resin-asphaltene) test findings.

Na_2_CO_3_ (99.9%), Ce (NO_3_)_3_.6H_2_O (98.5%), and Zn (NO_3_)_2_.6H_2_O (99%) were obtained from Merck company. All the purchased materials were used at a high level of qualifications.

### Methodology

#### Preparation of ZnO-Ce nanocomposites

The ZnO-Ce nanocomposites (95%Zn / 5%Ce) were prepared using the co-precipitation technique. 75 mL of Na_2_CO_3_ was added once 14.9 g of Zn (NO_3_) 2.6H_2_O and 1.2 g of Ce (NO_3_)_3_.6H_2_O had been dissolved in 100 mL of DW (distilled water). The solution was mixed at 70 °C for 2 h, filtered, washed, and dried at 80 °C for 12 h. A calcination process was conducted as the final step at 400 °C for 2 h to produce the nanocomposites.

#### Fluid properties measurements

**Figure 2:**
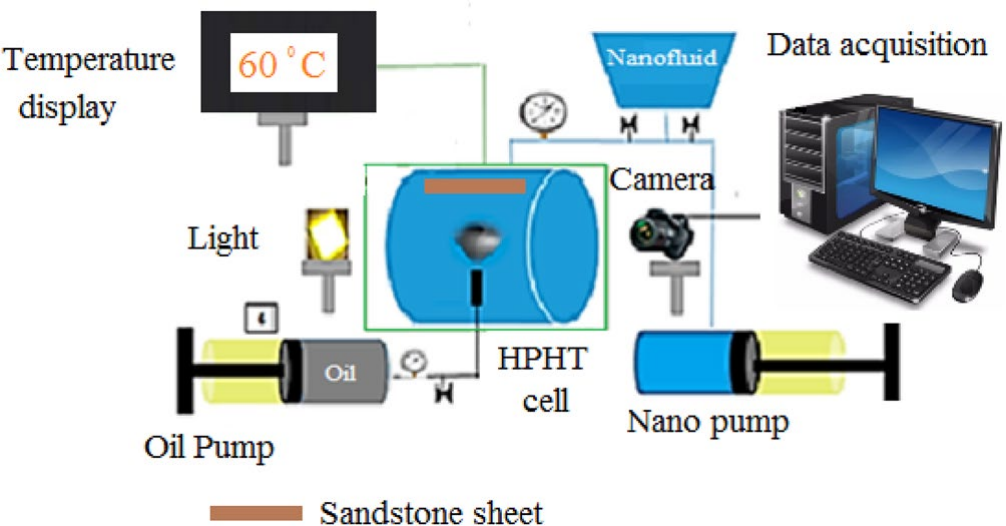
HPHT contact angle and interfacial tension set-up.

Since both the ZnO-cerium and ZnO nanoparticles were in powder form, 50,000 ppm formation brine and DW were used for preparing the nanofluids. Different concentrations including 25, 50, 75, 100, 125, and 150 ppm were designed to study the effect of the concentration on the nanofluid properties. In each concentration, the prepared fluid was stirred for 20 min. The zeta potential was measured using the Malvern Zetasizer Nano ZS ZEN3600 to confirm the nanofluid. The HPHT Vinci setup, as seen in Fig. 2, was used to measure the interfacial tension, and contact angle. For flooding with oil and water/nanofluid, there are two distinct pumps. Before closing the main cell, the trapped air was evacuated and the cell was filled with water (or nanofluid). The reservoir's temperature was kept at 60 °C and its pressure at 1800 Psi with the help of a heating jacket and water pump. A camera was used to take a picture of the oil droplet inside the water/nanofluid after it was released from the needle in Fig. 2 into the main cell. The data collected was then analyzed to determine the IFT. A sandstone sheet was put up to measure the contact angle. The same method and conditions were used to measure the contact angle of an oil droplet on the rock in the presence of water or nanofluid at 1800 Psi and 60 °C. ZnO and ZnO-cerium nanocomposites were present during IFT and CA experiments at 25, 50, 75, 100, 125, and 150 ppm at 1800 Psi and 60 °C. In this study, all experiments were conducted three times, and average values were given.

#### Coreflooding experiment

**Figure 3:**
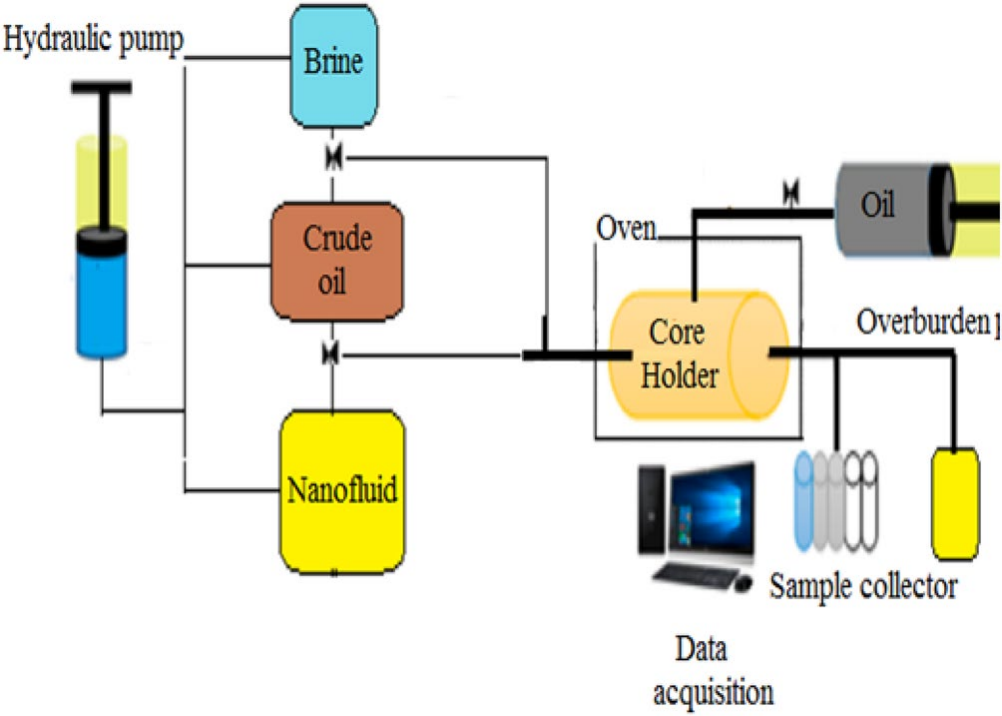
The core flooding set-up for the nanocomposites flooding process.

Figure 3 shows a schematics of Vinci core flooding apparatus for injecting nanocomposites into the sandstone core samples at 1800 Psi and 60 °C. Brine, crude oil, and nanofluid (ZnO-Ce or ZnO) were accumulated at three different transfer vessels. An oven was used for setting a uniform and constant temperature of 60 °C. Other main parts of the set-up were a sample collector at outlet for obtaining samples volume and calculating RF, overburden pump for removing air between rubber sleeve and sandstone core, core holder, and different pressure and differential pressure (DP) gauges. The sandstone core was vacuumed and saturated with crude oil, then aged at 1800 Psi and 60 °C for three weeks. The aged core was put in a core holder at 2000psi overburden pressure. 50,000 ppm brine was used for making nanofluid at optimum concentration of 100 ppm, and it was injected during water flooding for obtaining both nanocomposites effects on EOR at same reservoir conditions, 2000 Psi and 60 °C. Ultimate oil recovery, DP, and cumulative water volume were measured and reported.

## Results and discussion

### Chemical composition, surface morphology, and nanocomposites' characteristics

**Figure 4:**
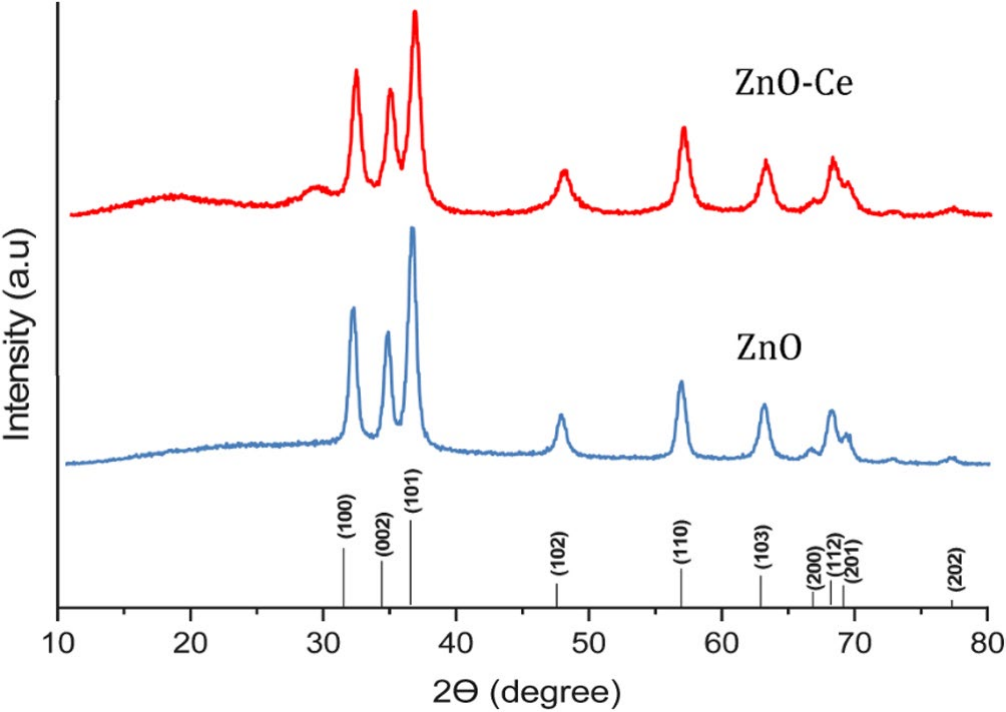
ZnO-cerium nanocomposite and ZnO XRD.

**Figure 5:**
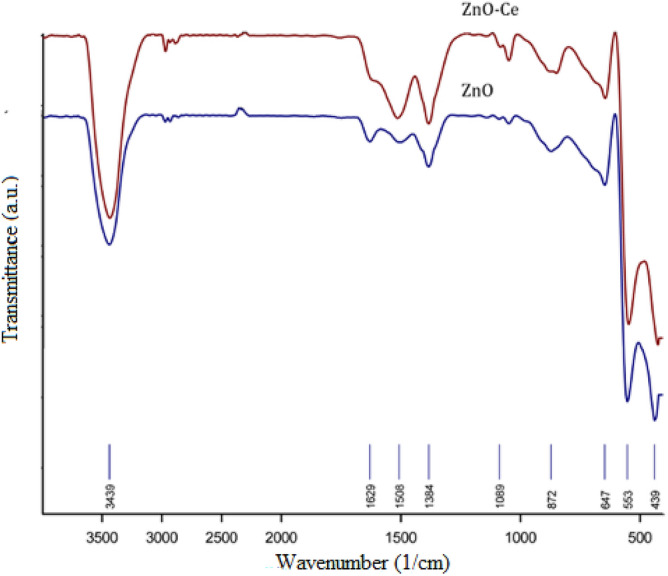
ZnO-cerium nanocomposites FTIR.

Figure 4 shows the XRD pattern of the prepared zinc oxide-cerium nanoparticle. A peak at 2θ = 29.2° shows the presence of zinc oxide-cerium (111 crystal facet) as a new phase^37^, and the following values are in conformity with the standard pattern of hexagonal wurtzite ZnO with JCPDS No. 36–1451: 30.15°, 33.95°, 37.16°, 47.21°, 56.92°, 63.21°, 67.1°, 68.13°, 69.12°, and 77.28°. Because Ce^3+^ has a wider ionic radius than Zn^2+^, most of the Ce ions are unable to be integrated into the ZnO lattice and are instead distributed as Ce_2_O_3_ and CeO_2_ on the ZnO surface^38^. Figure 5 compares the FTIR of zinc oxide-cerium nanocomposites to that of pure zinc oxide. Zn–O vibration is seen at wavelengths of 549 cm^−1^ and 553 cm^−1^^39^. The O–H group was also found in the 3180–3690 cm^−1^ range. Organic pollutants such as C‒O‒C, C=O, and C‒H were discovered at the 1000‒1100, 1300‒1650, and 2900‒3000 cm^−1^ bands, respectively, which is similar to what Rajbongshi et al.^40^ found. According to He et al.^38^, Zn–O and Ce–O correspond with one another, but Ce and N did not exhibit any peaks because of the little amount of dopants.

**Figure 6:**
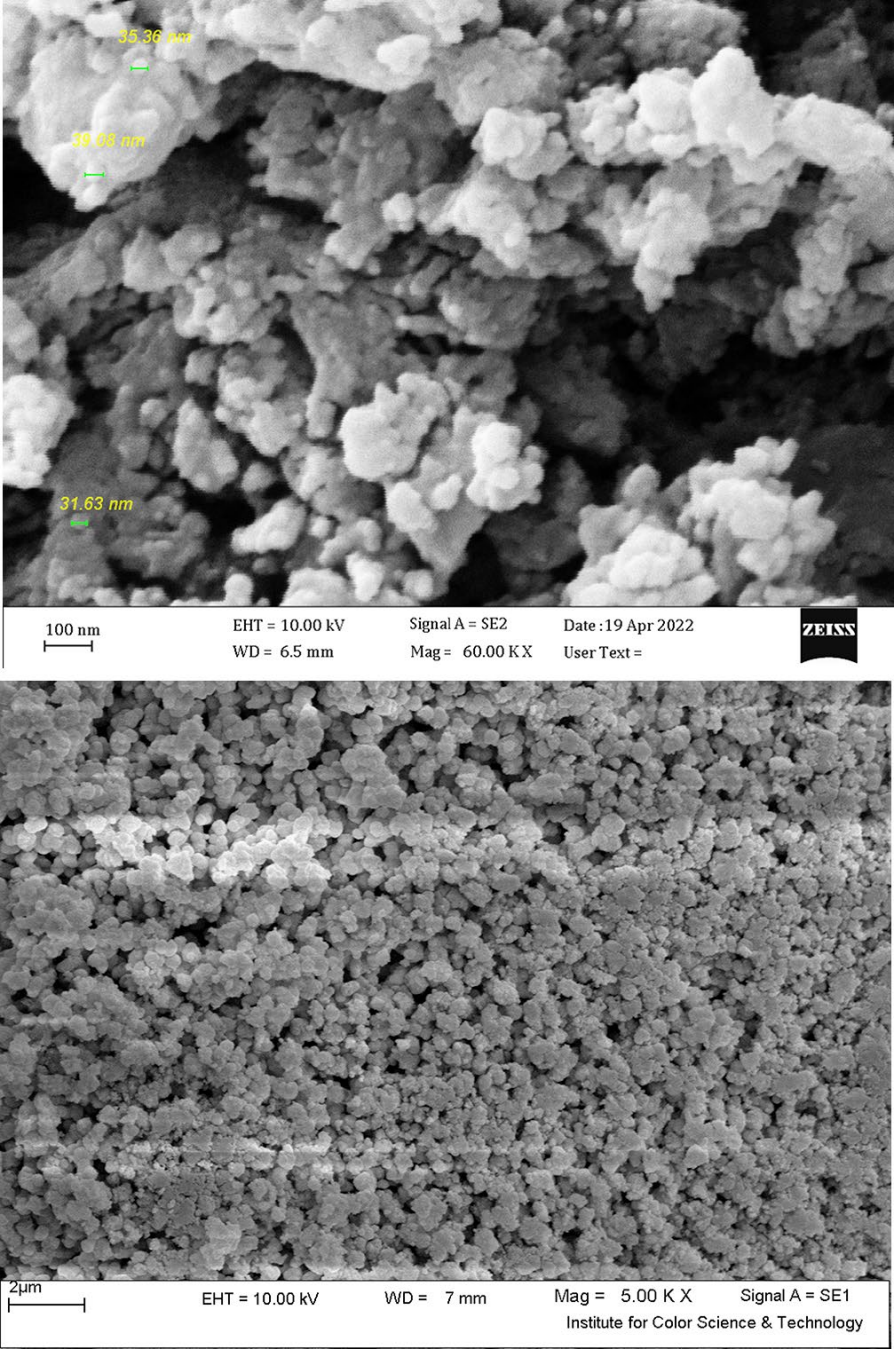
ZnO-cerium nanocomposites SEM nanocomposites at two different magnification (5kX and 60 kX).

**Figure 7:**
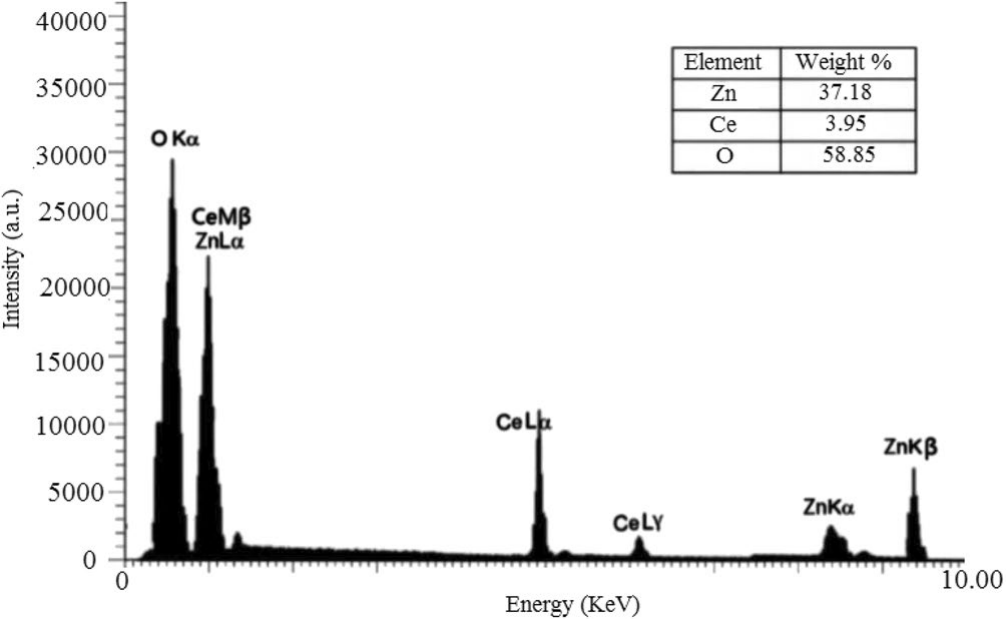
ZnO-cerium nanocomposites EDX.

Figures 6 and 7 show the nanocomposites SEM and EDX results, respectively. Based on the SEM tests, the nanocomposites are sphere-like, with a particle size of 20‒50 nm. The spectrum of EDX tests shows the presence of cerium, oxygen, and Zn. The Ce:Zn weight ratio was measured as 3.95:37.18.

**Figure 8:**
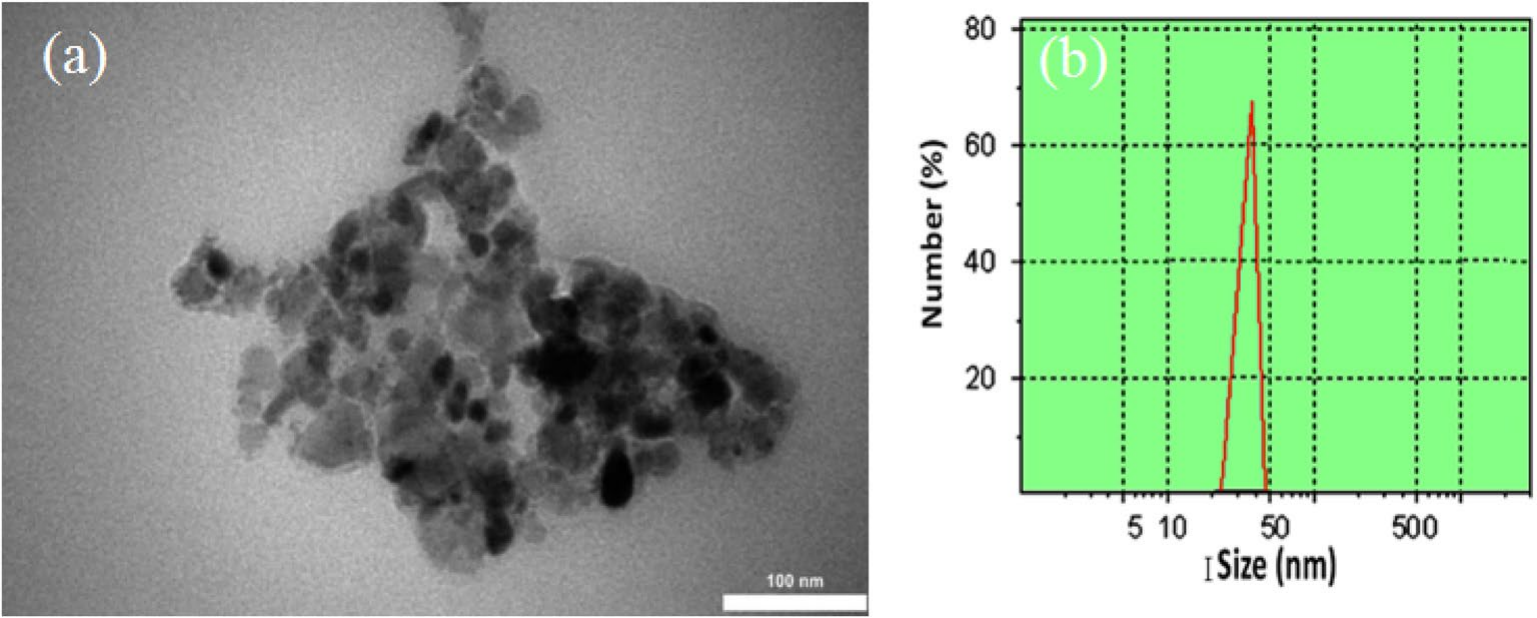
(**a**) TEM image and (**b**) DLS of Zn-Ce oxide NPs.

The ZnO-cerium nanocomposites' TEM data are displayed in Fig. 8a. The size was less than 50 nm and the shape was semi-spherical, consistent with the SEM data. The hydrodynamic size of the Zn-Ce oxide NPs was then evaluated by dynamic light scattering (DLS) measurements, where a distribution from 25 to 50 nm was observed (Fig. 8b). This value is entirely consistent with the results obtained from it the TEM analysis.

**Figure 9:**
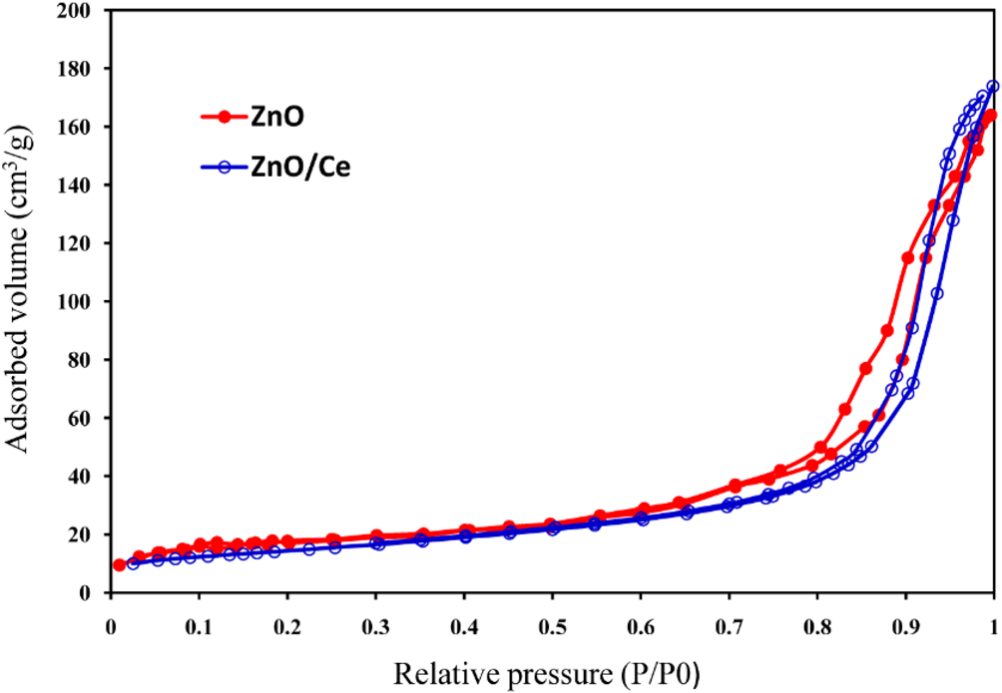
N_2_ adsorption–desorption by ZnO and ZnO-Ce samples.

The N_2_ adsorption–desorption isotherms of ZnO and ZnO-Ce samples using the Brunauer–Emmett–Teller (BET) method are shown in Fig. 9. The BET surface area, mean pore diameter, and pore volume are displayed in Table 1. The ZnO and ZnO-Ce samples have specific surface areas of 46.16 and 48.05 m^2^/g, respectively. The ZnO-Ce nanocomposites' pore volume rose when cerium was added to the ZnO. Following the addition of Ce to ZnO, the calcination process led to a gas release that increased the specific surface area. The effectiveness of ZnO-Ce as an EOR agent is impacted by this characteristic.

**Table 1:** Properties of ZnO and ZnO-Ce nanocomposites' textures.

Sample	Surface area (m^2^/g) ± SD	Pore volume (cm^3^ (STP) /g) ± SD	Mean pore diameter (nm) ± SD
ZnO	46.16 ± 1.03	8.98 ± 0.42	21.2 ± 0.87
ZnO/Ce	48.05 ± 0.74	10.75 ± 0.51	19.9 ± 0.77

SD: Standard deviation.

### Nanofluid specifications

The IFT, pH, density, viscosity, zeta potential (Z-P), and viscosity of nanofluid at different concentrations were measured as shown in Table 2.

**Table 2:** Nanofluid properties at different ZnO-cerium nanocomposites concentrations.

	Nanoparticlesconcentration (ppm)	Interfacial tension (mN m^-1^) ± SD	pH (-) ± SD	Density (g/cc) ± SD	Viscosity (cP) ± SD	Z-P (mV) ± SD	CA (°) ± SD
N-composites	25	39.60 ± 1.21	7.45 ± 0.17	0.9991 ± 0.001	1.91 ± 0.01	− 36.11 ± 1.54	156.14 ± 4.93
	50	35.11 ± 1.32	7.41 ± 0.32	0.9992 ± 0.004	1.94 ± 0.02	− 38.31 ± 1.37	96.38 ± 3.42
	75	31.15 ± 1.09	6.72 ± 0.16	0.9994 ± 0.003	1.97 ± 0.03	− 40.31 ± 1.75	85.18 ± 4.66
	100	22.51 ± 1.10	6.45 ± 0.14	0.9995 ± 0.001	1.98 ± 0.02	− 44.36 ± 2.03	40.83 ± 3.98
	125	15.48 ± 0.98	6.58 ± 0.09	0.9996 ± 0.002	2.00 ± 0.03	− 39.50 ± 2.25	51.16 ± 4.54
	150	12.24 ± 1.02	7.06 ± 0.75	0.9998 ± 0.002	2.02 ± 0.03	− 36.11 ± 1.72	55.21 ± 3.27
ZnO	25	54.25 ± 2.81	6.71 ± 0.32	0.9990 ± 0.003	1.70 ± 0.03	− 25.28 ± 1.38	170.37 ± 5.63
	50	49.12 ± 2.34	6.67 ± 0.16	0.9991 ± 0.001	1.73 ± 0.06	− 26.82 ± 1.20	116.39 ± 4.95
	75	42.60 ± 1.88	6.05 ± 0.66	0.9992 ± 0.002	1.75 ± 0.04	− 28.22 ± 1.34	103.3 ± 4.04
	100	30.50 ± 1.21	5.81 ± 0.54	0.9993 ± 0.005	1.76 ± 0.06	− 31.05 ± 1.86	50.21 ± 3.22
	125	21.68 ± 1.18	5.92 ± 0.76	0.9995 ± 0.001	1.78 ± 0.05	− 27.65 ± 1.49	62.48 ± 3.63
	150	17.18 ± 0.93	6.35 ± 0.59	0.9999 ± 0.002	1.80 ± 0.06	− 25.28 ± 1.27	67.30 ± 4.15

SD: Standard deviation.

**Figure 10:**
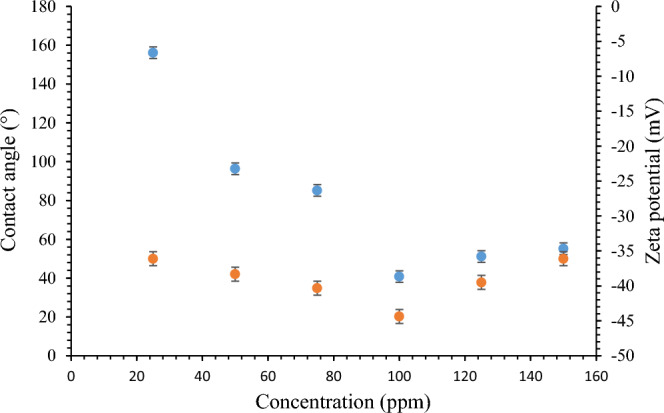
Selecting an optimum nanocomposites concentration according to CA and stability tests.

Figure 10 demonstrates the amount of wettability and zeta potential at different nanofluid concentrations. The absolute quantity of zeta potential tests showed a balanced between the electrostatic repulsive and attractive forces^41–43^. Hence, the zeta potential of ˃ ± 30 mV shows high repulsive forces between nanoparticles and a stable nanofluid. The most negative zeta potential value was observed at 100 ppm, which was -44.36 mV. The maximum reduction in CA and a shift to a more water-wet state were also observed at the same concentration. Hence, 100 ppm was selected as the most compelling case for the oil displacement test. Based on the findings presented in Table 2, the optimum concentration of ZnO was observed at 100 ppm with consideration of the CA and zeta potential tests, and this was selected as a base case to be compared to our new approach.

### Displacement tests

**Figure 11:**
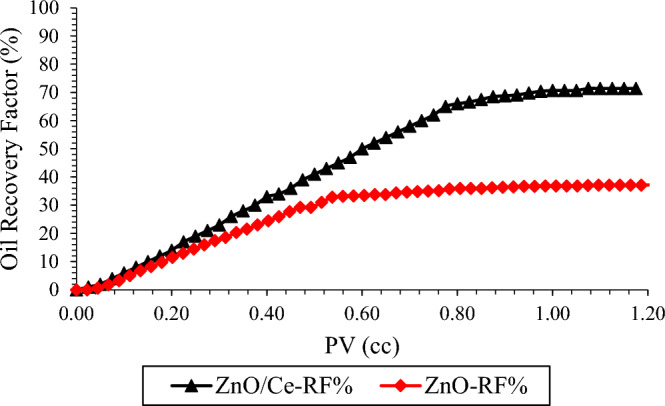
Comparison of RF in the presence of N-composites and ZnO nanoparticles.

Figure 11 showsthe recovery factor achieved by injecting the nanocomposites at OPC. ZnO-cerium nanocomposites had higher recovery than the base case (pure ZnO), and the RF% increased from 37.11% to 71.40% at 60 °C and 1800 Psi. The main reason can be found in Table 2. According to the results, the nanocomposites were more stable and effective in altering the IFT and wettability at all concentrations. For example, at OPC, [IFT (mN/m), CA (°), Z-P (mV)] are (22.51, 40.83, − 44.36) and (30.50, 50.21, − 31.05) for nanocomposites and ZnO nanoparticles, respectively.

**Figure 12:**
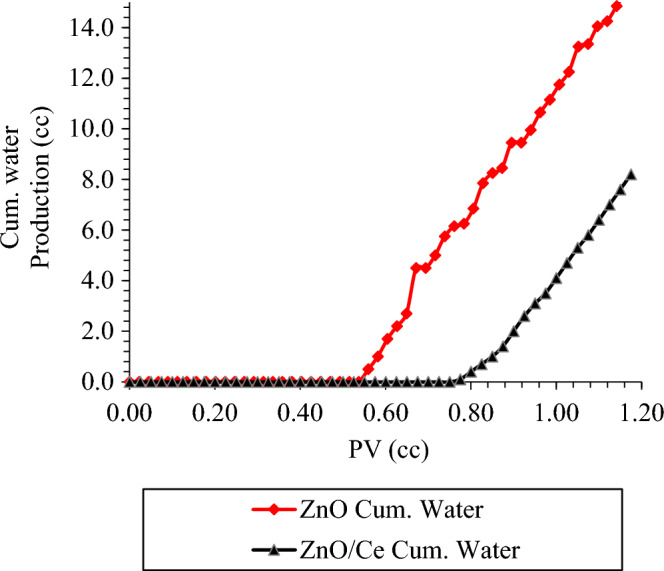
Comparing the total amount of water produced when ZnO nanoparticles and N-composites are present.

Figure 12 shows the cumulative water production of nanocomposites at 100 ppm, 1800 Psi, and 60 °C. Based on the graph, the water breakthrough was delayed by the modified nanocomposites flooding. This delay shows the change in macroscopic sweep efficiency and a more invasion to the smaller pores. Hence, the application of ZnO-Ce nanofluid results in more uniform flow compared to ZnO nanofluid.

**Figure 13:**
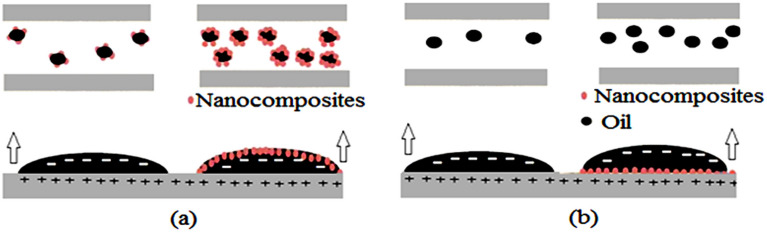
Main nanocomposites mechanisms **a**) adsorption of oil in nanocomposites surface, **b**) adsorbing nanocomposites on the surface of the rock.

Wettability alteration, and zeta potential were two main factors for getting higher tertiary oil recovery when nanocomposites are present. As said at OPC = 100 ppm, contact angle for ZnO-Cewas lower compared to pure ZnO, and ZnO-Ce was more stable in compared to ZnO. Figure 13a, b were shown effects of wettability alteration in adsorption of oil in nanocomposites surface, separation of nanocomposites, and adsorbing on the surface of the rock, and switching, correspondingly, from oil-to-water-wettability. Another main mechanisms for obtaining higher efficiency of ZnO-Ce was obtaining higher IFT reduction in compared to pure ZnO. Finally, lower absolute permeability reduction were observed in the presence of ZnO-Ce (9.2%) in compared to pure ZnO (19.2%). Since all conditions were same, higher incremental oil recovery was logical due to above two mechanisms.

## Conclusion

This study examined the characteristics of ZnO-Cerium nanocomposites with ZnO nanoparticles using XRD, BET, EDX, SEM, TEM, DLS, and FT-IR. The addition of cerium to the Zinc oxide increased the pore volume of the Zinc-Cerium nanocomposites. The calcination procedure that followed adding Ce to ZnO increased the specific surface area by causing a gas release. The IFT, CA, and Z-P experiments were used to determine the ideal concentration of nanocomposites to have the most impact on the interactions between rock, oil, and brine. At 100 ppm, the most stable nanofluid was developed which altered the wettability and viscosity the most. Moreover, same static tests were performed for ZnO nanoparticles, and based on the results, ZnO-Cerium IFT and CA reduction are better impacted by nanocomposites, and better stability compared to ZnO nanoparticles. Based on the core flooding tests at 1800 Psi, 60 °C, and OPC = 100 ppm. The recovery factor improved to 71.40% from 37.11%. Due to adding cerium in nanocomposites. Moreover, the water breakthrough was delayed by the modified nanocomposites flooding in compared to ZnO nanoparticles.

## Data Availability

The corresponding author can provide the datasets used and/or analyzed for this study upon reasonable request.
